# Identification of tomato plant as a novel host model for *Burkholderia pseudomallei*

**DOI:** 10.1186/1471-2180-10-28

**Published:** 2010-01-29

**Authors:** Yian Hoon Lee, Yahua Chen, Xuezhi Ouyang, Yunn-Hwen Gan

**Affiliations:** 1 Department of Biochemistry, Yong Loo Lin School of Medicine, National University of Singapore, 8 Medical Drive, 117597, Singapore; 2 Immunology Program, National University of Singapore, 28 Medical Drive, 117456, Singapore; 3 Microscopy and Imaging Facilities, Temasek Life Sciences Laboratory, 1 Research Link, 117604, Singapore

## Abstract

**Background:**

*Burkholderia pseudomallei *is the causative agent for melioidosis, a disease with significant mortality and morbidity in endemic regions. Its versatility as a pathogen is reflected in its relatively huge 7.24 Mb genome and the presence of many virulence factors including three Type Three Secretion Systems known as T3SS1, T3SS2 and T3SS3. Besides being a human pathogen, it is able to infect and cause disease in many different animals and alternative hosts such as *C. elegans*.

**Results:**

Its host range is further extended to include plants as we demonstrated the ability of *B. pseudomallei *and the closely related species *B. thailandensis *to infect susceptible tomato but not rice plants. Bacteria were found to multiply intercellularly and were found in the xylem vessels of the vascular bundle. Disease is substantially attenuated upon infection with bacterial mutants deficient in T3SS1 or T3SS2 and slightly attenuated upon infection with the T3SS3 mutant. This shows the importance of both T3SS1 and T3SS2 in bacterial pathogenesis in susceptible plants.

**Conclusions:**

The potential of *B. pseudomallei *as a plant pathogen raises new possibilities of exploiting plant as an alternative host for novel anti-infectives or virulence factor discovery. It also raises issues of biosecurity due to its classification as a potential bioterrorism agent.

## Background

*Burkholderia pseudomallei *is a Gram-negative bacterium that is the causative agent for melioidosis, a disease endemic in Southeast Asia and Northern Australia with significant morbidity and mortality [1,2]. The bacterium exhibits broad host range and has been shown to cause disease in cattle, pigs, goats, horses, dolphins, koalas, kangaroos, deers, cats, dogs and gorillas [3]. Acquisition of the bacterium could be through inhalation of aerosol, ingestion of contaminated water and inoculation through open skin [4]. In humans, the disease could present with varied manifestations ranging from asymptomatic infection, localized disease such as pneumonia or organ abscesses to systemic disease with septicemia [5]. The disease could be acute or chronic, and relapse from latency is possible [6].

The versatility of *B. pseudomallei *as a pathogen is reflected in its huge 7.24 Mb genome organized into two chromosomes [7]. One of the most important virulence factors that has been partially characterized in *B. pseudomallei *is its Type Three Secretion Systems (T3SS), of which it has three [8,9]. Each T3SS typically consists of a cluster of about 20 genes encoding structural components, chaperones and effectors which assemble into an apparatus resembling a molecular syringe that is inserted into host cell membrane for the delivery of bacterial effectors into host cell cytosol. One of the *B. pseudomallei *T3SS known as Bsa or T3SS3 resembles the *inv/mxi/spa *T3SS of *Salmonella *and *Shigella*, and has been shown to be important for disease in animal models [10]. The other two T3SS (T3SS1 and 2) resemble the T3SS of plant pathogen *Ralstonia solanacearum *[11] and do not contribute to virulence in mammalian models of infection [12]. Being a soil saprophyte and having the plant pathogen-like T3SS raise the possibility that *B. pseudomallei *could also be a plant pathogen. As *B. pseudomallei *is a risk group 3 agent with specific requirements for containment, we first test this hypothesis using the closely related species *B. thailandensis *as a surrogate model especially in experiments where risk of aerosolization is high, before we verify key experiments with *B. pseudomallei*. *B. thailandensis *is considered largely avirulent in mammalian hosts unless given in very high doses [13,14]. We infected both tomato as well as rice plants with *B. pseudomallei *to determine their susceptibility to disease. Furthermore, the role of the three *B. pseudomallei *T3SS in causing plant disease is evaluated and the implication of the ability of *B. pseudomallei *to infect plants is discussed.

## Methods

### Bacterial strains, plasmids and growth conditions

All bacterial strains, plasmids used and constructed are listed in Table 1. All strains of *B. thailandensis *and *B. pseudomallei *were cultured at 37°C in Luria-Bertani (LB) medium or on Tryptone Soy Agar (TSA) plates. To obtain log-phase culture, 250 μL of overnight culture was inoculated into 5 mL LB medium and cultured for 2.5 hours with constant shaking at 100 rpm. *Escherichia coli *strains were cultivated at 37°C in LB medium. Antibiotics were added to the media at the following final concentrations of 100 μg/mL (ampillicin); 25 μg/mL (kanamycin); 10 μg/mL (tetracycline); and 25 μg/mL (zeocin) for *E. coli*, 250 μg/mL (kanamycin); 40 μg/mL (tetracycline); 25 μg/mL (gentamicin) and 1000 μg/mL (zeocin) for *B. pseudomallei*. All antibiotics were purchased from Sigma (St Louis, MO, USA).

**Table 1:** All bacterial strains, plasmids used and constructed.

Name	Description	Source or Reference
pK18*mobsac*B	oriT; Km^R^; *sacB *gene	[32]
pGEM-tet	pGEM containing a tetracycline resistance cassette, Tet^R^, Amp^R^	Y. Chen, unpublished
pCLOXZ1	pGEM containing a zeocin resistance cassette, Zeo^R^, Amp^R^	Y. Chen, unpublished
pT3SS1/upstream/downstream/tet	pK18*mobsac*B containing upstream and downstream of TTSS1 flanking a tet cassette, Km^R^, Tet^R^	This study
pT3SS2/upstream/downstream/tet	pK18*mobsac*B containing upstream and downstream of TTSS2 flanking a tet cassette, Km^R^, Tet^R^	This study
pT3SS3/upstream/downstream/zeo	pK18*mobsac*B containing upstream and downstream of TTSS3 flanking a zeo cassette, Km^R^, Zeo^R^	This study
*E. coli*		
DH5α	Infection strain	Lab stock
TG1	Cloning host	Zymo Research
SM10λpir	Conjugation strain	[33]
*B. thailandensis*		
ATCC700388		ATCC
*B. pseudomallei*		
K96243	Clinical isolate	Thailand
561	Kangaroo isolate	Eu Hian Yap, unpublished
612, 490	Avian isolates	Eu Hian Yap, unpublished
77/96, 109/96	Soil isolates	Eu Hian Yap, unpublished
KHW	Wild-type parental strain, clinical isolate, Km^S^	[20]
KHWΔT3SS1	BPSS1386-1411 region was replaced with tet cassette, Tet^R^, Km^S^	This study
KHWΔT3SS2	BPSS1592-1629 region was replaced with tet cassette, Tet^R^, Km^S^	This study
KHWΔT3SS3	BPSS1520-1552 region was replaced with zeo cassette, Zeo^R^, Km^S^	This study

### Plant material

Tomato seeds of the *Solanum lycopersicum *variety Season Red F1 Hybrid (Known-You Seeds Distribution (S.E.A) Pte Ltd) and *Arabidopsis thaliana *(Loh Chiang Shiong, NUS) were surface sterilized with 15% bleach solution for 15 minutes with vigorous shaking. The seeds were rinsed in sterile distilled water and germinated in MS agar medium. The seedlings were cultivated with a photoperiod of 16 hour daylight and 8 hour darkness. One month old plantlets were used for infection. Tomato plantlets were transferred into 50 mL Falcon tubes with 5 mL of liquid MS medium for infection while 1 mL of MS medium was used for Arabidopsis. Rice seeds (*Japonica nipponbare*) were obtained from Dr Yin Zhong Zhao (Temasek Life Sciences Laboratories, Singapore). Seeds were surface sterilized as described above. The seeds were rinsed in sterile distilled water and germinated in N6 agar medium. The germinated seedlings were placed on N6 agar supplemented with 2 mg/mL of 2, 4-dichlorophenyoxyacetic acid (2, 4-D) in the dark to induce callus production. The callus were regenerated on N6 medium supplemented with 2 mg/mL Benzylaminopurine (BA), 1 mg/mL Naphthylacetic Acid (NAA), 1 mg/mL Indole-3-acetic acid (IAA) and 1 mg/mL Kinetin under 16 hour daylight and 8 hour dark photoperiod. Rice plantlets were transferred and maintained in MS agar medium. The plantlets were transferred into 50 mL Falcon tubes with 5 mL of liquid MS medium for infection. Some plantlets were also wounded by cutting off the roots before being transferred.

### Plant infection

Tomato, rice and Arabidopsis plantlets were infected with log phase cultures at the concentration of 1 × 10^7 ^colony forming units (cfu)/5 mL medium by immersing only the roots of the plantlets in the inoculum in a 50 mL tube. The plantlets were maintained at 24-25°C, shaking at 100 rpm. The plantlets were observed for symptoms such as yellowing of leaves, blackening of the leaf veins, wilting and necrosis daily over 7 days. Each plantlet was scored daily on a disease index score of 1 to 5 based on how extensive the symptoms were as calculated by the percentage of the plant with symptoms (1: no symptoms; 2: 1 to 25% of the plant showed symptoms; 3: 26 to 50% of the plant showed symptoms; 4: 51 to 75% of the plant showed symptoms; 5: 76 to 100% of the plant showed symptoms or the plant was dead) [15]. Each experiment included at least 12 to 20 plantlets infected with bacteria except for experiments with rice and Arabidopsis plantlets where 6 plantlets were used. All experiments were repeated at least twice.

### Multiplication of *B. thailandensis *in tomato plantlets and leaves

Tomato plantlets were infected with bacteria through unwounded roots and three leaves from each plantlet were excised at day 1, 3, 5 and 7 after infection. The leaves were macerated in 1 mL PBS with a micro-pestle, serially diluted and plated on TSA plates in duplicates. Tomato leaves were infected by cutting with a pair of scissors dipped in 1 × 10^9 ^cfu/mL of *B. thailandensis*. Five plantlets were used in each experiment. At days 1 and 3 after infection, one infected leaf from each plantlet was excised, washed with 10% bleach solution for 1 min and rinsed with sterile water. The leaf was blotted dry on sterile filter paper and imprinted on TSA agar plates to determine if there were any bacteria on the surface of the leaves. The imprinted plates were incubated at 37°C for 24 hours before checking for any bacteria growth. The leaves were then weighed and macerated in 1 mL PBS with a micro-pestle, serially diluted and plated on TSA plates in duplicates. Only leaf samples which did not show any bacteria growth on the imprinted plates will be counted to avoid counting contaminating bacteria from leaf surfaces.

### Transmission Electron Microscope (TEM)

Tomato leaf and rice blade were infected by cutting with a pair of scissors dipped in 1 × 10^9 ^cfu/mL of *B. pseudomallei *strain KHW or *B. thailandensis*. One day after infection, the infected tomato leaf and rice blade were excised for TEM. One millimeter from the infected leaf/blade edge were cut and discarded to avoid contamination from extracellular bacteria at the infection site. A further two millimeter from the infected leaf/blade edge were then cut and sliced into smaller sections and fixed with 4% glutaraldehyde in 0.1 M phosphate buffer under vacuum for 4 hours. It was post-fixed with 1% osmium tetroxide in 0.1 M phosphate buffer for 1 hour at 4°C. Samples were dehydrated sequentially through 30%, 50%, 70%, 90%, 100% ethanol, and finally in propylene oxide prior to infiltration with Spurr resin [16]. Samples were embedded in 100% spur resin and polymerized at 70°C overnight. Ultra-thin sections were cut on a Leica Ultracut UCT ultra-microtome and examined with a transmission electron microscope (JEM1230, JEOL, Japan) at 120 kV.

### Growth of bacteria in different media

Overnight cultures were used to inoculate 5 mL of LB and Murashige and Skoog (MS) [17] medium to a starting optical density at 600 nm of 0.1. The cultures were incubated at 37°C for LB medium and 25°C for MS medium. Optical density at 600 nm for all cultures was measured at 0, 2.5, 6 and 24 hours. All experiments were repeated twice with duplicates.

### Generation of *B. pseudomallei *T3SS1, T3SS2 and T3SS3 mutants

Approximate one kb fragments upstream and downstream of the T3SS1, T3SS2 or T3SS3 locus were amplified from *B. pseudomallei *KHW genomic DNA and subsequently cloned into pK18*mobsacB*. The tet cassette from pGEM-tet or zeo cassette (kindly provided by Dr Herbert Schweizer, Colorado State University, USA) from pCLOXZ1 was inserted between the upstream and downstream fragments resulting in pT3SS1/upstream/downstream/tet, pT3SS2/upstream/downstream/tet, and pT3SS3/upstream/downstream/zeo. The plasmids were electroporated into SM10 conjugation host and conjugated into *B. pseudomallei *strain KHW. Homologous recombination was selected for retention of antibiotic marker (Tet or Zeo) linked to the mutation and loss of the plasmid marker (Km) to generate KHWΔT3SS1, KHWΔT3SS2 and KHWΔT3SS3. Each mutant was confirmed by PCR for the loss of a few representative T3SS genes in the locus.

### Cytotoxicity assay on THP-1 cells

Human monocytic cell line THP-1 were maintained in RPMI 1640 (Sigma), supplemented with 10% Fetal Calf Serum (FCS, Hyclone Laboratories, Logan, UT), 200 mM L-glutamine, 100 Unit/mL penicillin and 100 μg/mL streptomycin. THP-1 cells were seeded at a concentration of 1 × 10^6 ^cells per 100 μL in 96-well plate in medium without FCS and antibiotics. Log phase bacteria were used for infection at multiplicity of infection (MOI) of 100:1. Kanamycin (250 μg/mL) was added one hour after infection to suppress the growth of extracellular bacteria. Supernatant was collected 6 hours after infection. Lactate dehydrogenase (LDH) activity in the supernatant was measured with the Cytotoxicity Detection Kit (Roche) according to manufacturer's instruction. Percentage cytotoxicity was calculated by the formula:

### Statistical analysis

Average disease scores with standard deviation were calculated based on at least 100 tomato plantlets infected with each strain of bacteria or mutant. Data were analyzed using repeated measure analysis of variance [18]. All statistical analyses were performed using SPSS version 17 software (SPSS Inc). A *p *value of less than 0.001 is considered significant.

## Results

### Using *B. thailandensis *infection of tomato plantlets as a model

**Figure 1:**
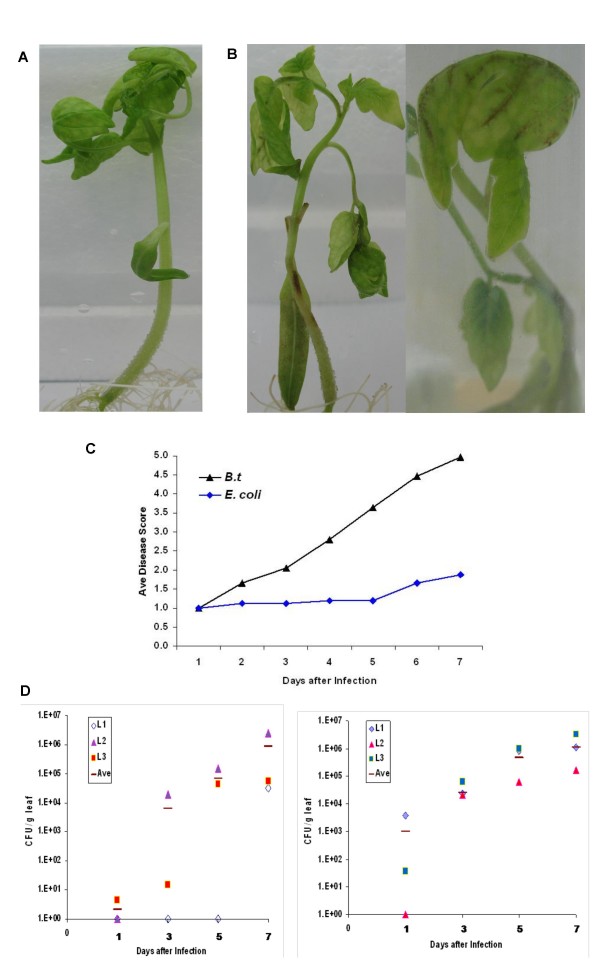
***B. thailandensis *infection and replication in tomato plantlets**. Tomato plantlets were infected with *B. thailandensis *and monitored over a period of seven days. On day 7, representative photographs of the uninfected plantlets (A) and the infected plantlets (B) were taken. (C) Tomato plantlets infected with *B. thailandensis *were scored daily based on the extent of disease symptoms on an index from 1 - 5 over a period of seven days. The average score was calculated based on at least 100 plantlets cumulative from several experiments. (D) Each graph represents bacterial counts from leaves of one *B. thailandensis *infected plantlet over days 1, 3, 5 and 7. Each symbol L represents one leaf.

To mimic infection via a possible natural route, the unwounded roots of tomato plantlets were immersed in media inoculated with 1 × 10^7 ^cfu of bacteria. Only the roots were in contact with the inoculum. Tomato plantlets infected via the roots by *B. thailandensis *showed progressive symptoms such as yellowing of leaves, blackening of the leaf veins, wilting and necrosis whereas uninfected plantlets remained healthy and did not show any disease symptoms throughout the period (Fig 1A-B). Most infected plantlets were dead on day 7. All plantlets were monitored over a period of seven days. Disease was scored daily for every plantlet on an index from 1-5 based on the extent of symptoms presented as described in Methods. The average disease score for a particular day represent the mean disease scores for all the plantlets with the same treatment on that day. As infection progressed over time, the average disease score for *B. thailandensis*-infected plants increased progressively, reaching a maximum disease score of 5 on day 7 (Fig 1C). In contrast, plantlets infected with *E. coli *in the same manner via the roots showed a slight progression of average disease scores over time and reached a maximum disease score of 2 on day 7 (Fig 1C), demonstrating that the extensive disease and death seen was specific to *B. thailandensis *infection and not due to non-specific stress induced by the experimental manipulations.

For a phytopathogen to successfully colonize the plant, it must be able to replicate intercellularly [19]. To determine whether bacteria are able to replicate intercellularly, we sampled leaves from two representative plantlets which had been inoculated with bacteria via unwounded roots at 1, 3, 5 and 7 days post-inoculation. Three leaves were sampled at each time-point per plantlet. Both plantlets showed a progressive increase in bacterial load in their leaves over time (Fig 1D).

### Susceptibility of tomato plantlets to *B. pseudomallei *infection

**Figure 2:**
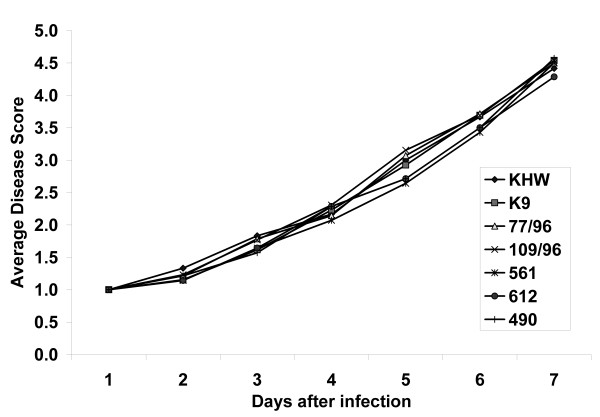
**Infection of tomato plantlets with different *B. pseudomallei *isolates**. KHW and K9 (K96243) are clinical isolates, 77/96 and 109/96 are soil isolates, 561 is isolated from a kangaroo, 612 from a crown pigeon and 490 from a Bird of Paradise. The average disease score was calculated based on 12 plantlets per bacterial isolate cumulative from two experiments.

Having established that *B. thailandensis *can infect tomato plantlets and cause disease, we determine whether *B. pseudomallei *would similarly infect tomato plantlets. We included strains isolated from humans, animals or the environment such as two clinical isolates (K96243 and KHW), a kangaroo isolate 561, two bird isolates (612 and 490) and two soil isolates (77/96 and 109/96) on their ability to infect tomato plants. *B. pseudomallei *is able to infect tomato plantlets to a similar degree as *B. thailandensis *with almost identical disease symptoms. All isolates were able to infect and cause disease to a similar extent (Fig 2), showing that the ability to infect susceptible plants is unlikely to be strain-specific.

### Localization of bacteria at site of infection

**Figure 3:**
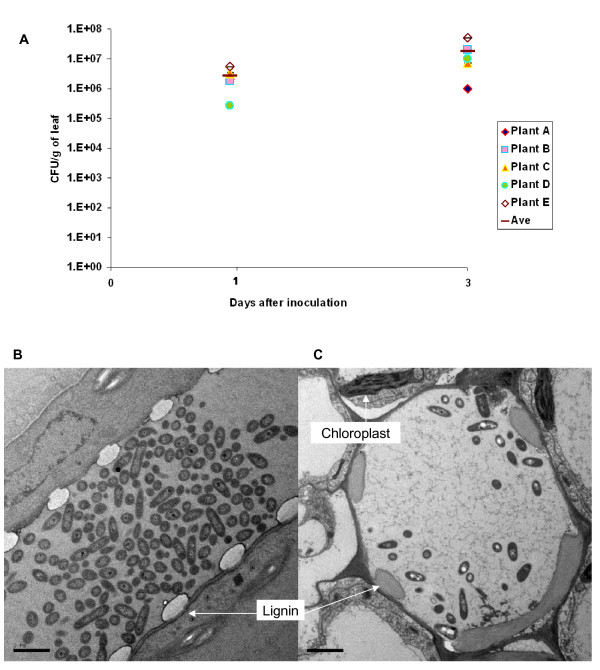
**Replication and localization of bacteria in tomato leaves**. A) *B. thailandensis *multiplication in tomato leaves was measured at one and three days post inoculation. The graph is representative of two separate experiments. Representative transmission electron micrographs show localization of bacteria in tomato leaf determined one day after infection. B) Leaves infected with *B. thailandensis *showing the longitudinal section of xylem vessel and C) leaves infected with *B. pseudomallei *showing the cross-sectional view. Bar represents 2 μm.

Having established the ability of both *B. thailandensis *and *B. pseudomallei *to be phytopathogens capable of infecting tomato plants, we next examined the localization of the bacteria upon inoculation into the leaf via TEM. We first examined whether bacteria inoculated into the leaves were able to survive and replicate. To ensure that there were no bacteria on the leaf surfaces, the leaves were surface sterilized with bleach and washed in sterile water before weighing and maceration. *B. thailandensis *was able to replicate in the leaves after inoculation (Fig 3A). The number of bacteria increased by about 10 fold three days after infection although the numbers did not reach statistical significance by the student *t *test (p > 0.05). When examined under TEM, *B. pseudomallei *and *B. thailandensis *could be found in the xylem of the vascular bundle of the inoculated leaf (Fig 3B-C). The rest of the surrounding cells were not colonized, suggesting that the bacteria spread to the rest of plant through the xylem vessels.

### The role of T3SS in plant infection

**Figure 4:**
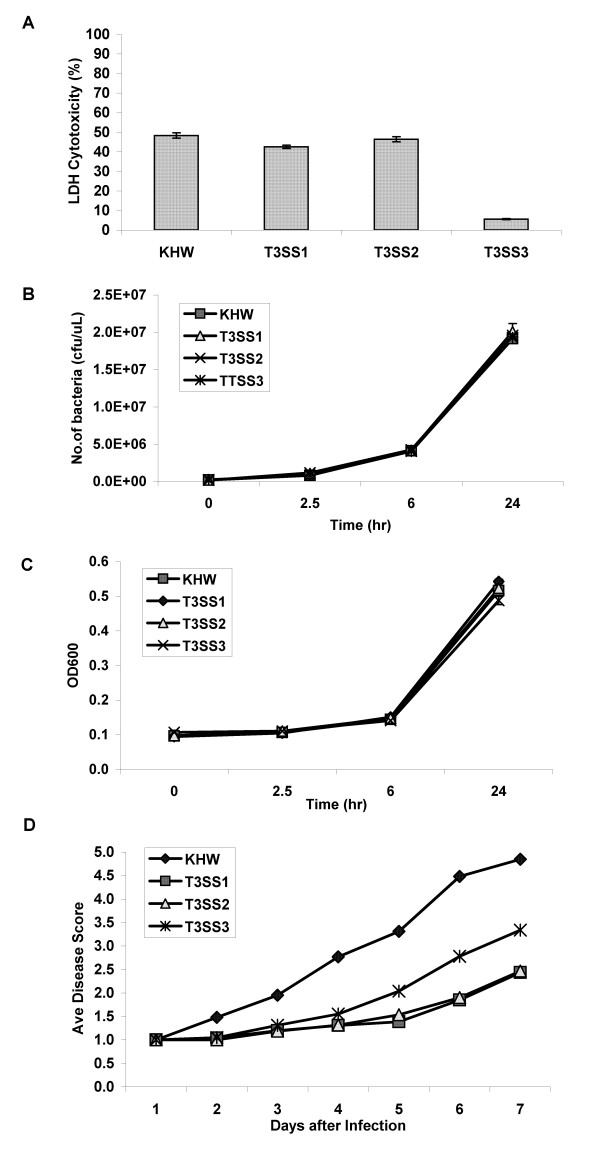
**The role of T3SS in plant infection**. (A) Cytotoxicity of wild-type *B. pseudomallei *and its T3SS mutants on THP-1 cells infected for six hours at an MOI of 100:1. Growth of *B. pseudomallei *and its T3SS mutants in LB (B) and MS (C) media. The graph is representative of two separate experiments. (D) Virulence of wildtype *B. pseudomallei *and its T3SS mutants on tomato plantlets. The average disease score with standard deviation is calculated based on at least 100 plantlets cumulative from several experiments.

To determine the role of T3SS in plant infection, we created *B. pseudomallei *deletion mutants lacking the entire region of T3SS1, T3SS2 or T3SS3 in strain KHW (Table 1). We first examined these mutants in the established macrophage cytotoxicity model and confirmed the necessity of T3SS3 in mediating cytotoxicity [20] whereas mutants losing T3SS1 and T3SS2 were as cytotoxic as wildtype bacteria to THP-1 cells (Fig 4A). This shows that T3SS1 and T3SS2 are not involved in mediating cytoxicity to mammalian cells. To exclude the possibility that any defect we see with the T3SS mutants would be due to a reduced fitness, we ascertained that all mutants grew as well as wildtype bacteria in LB and plant MS medium (Fig 4B-C). However, infection of tomato plantlets via unwounded roots showed that plants infected by the T3SS1 and T3SS2 mutants exhibited significant delay in disease compared to plants infected by wildtype bacteria (Fig 4D). Statistical analysis of the average disease score over 7 days showed that the T3SS1, 2 and 3 mutants were significantly less virulent from the wildtype bacteria (*p *< 0.001). T3SS1 and T3SS2 mutants were also significantly less virulent compared to the T3SS3 mutant (*p *< 0.001). This shows that both T3SS1 and T3SS2 contribute significantly to pathogen virulence towards tomato plants. The T3SS3 mutant also showed an intermediate degree of virulence between wildtype bacteria and the T3SS1 and T3SS2 mutants, likely because T3SS3 has a non-redundant role in mediating virulence in the susceptible tomato plants.

### Susceptibility of rice and Arabidopsis plantlets to *B. pseudomallei *and *B. thailandensis *infection

**Figure 5:**
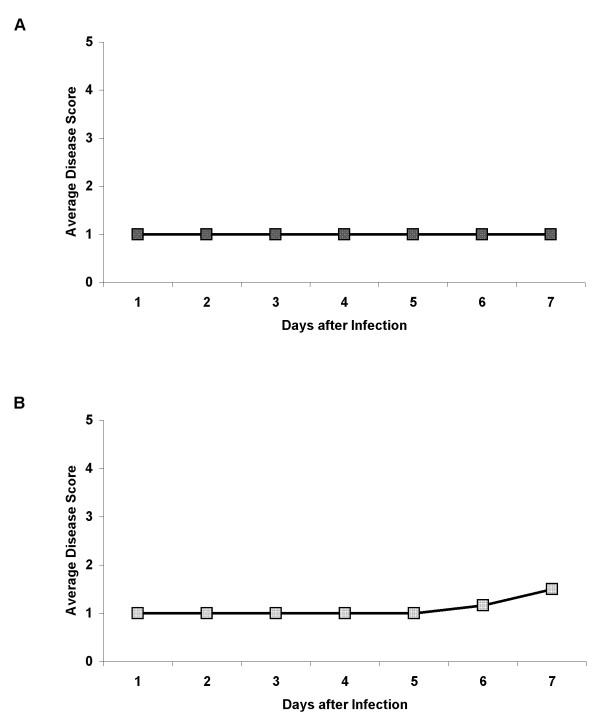
***B. pseudomallei *and *B. thailandensis *infection of rice (A) and *Arabidopsis *(B) plantlets**. Each graph represents an experiment of 6 plantlets infected either with *B. pseudomallei *or *B. thailandensis *as both types of infections resulted in identical disease scores. Each experiment with *B. pseudomallei *or *B. thailandensis *infection had been repeated twice.

Both *B. thailandensis *and *B. pseudomallei *did not cause any discernible symptoms in rice plantlets when infected via roots (unwounded or wounded) nor via inoculation through the leaves. *B. thailandensis *and *B. pseudomallei *infection of rice plantlets showed identical disease scores over 7 days (Fig 5A). We were unable to recover any bacteria from the leaves after infection via the roots. When bacteria were inoculated directly into the leaf blade, no bacteria were recoverable from the leaf one day after inoculation, indicating a lack of establishment of infection. The inoculated leaves did not show any yellowing (data not shown) as seen in the tomato leaves. Thus, rice plants are non-hosts to the bacteria. As *Arabidopsis thaliana *has been used extensively as a plant host model for several pathogens, we tested *B. thailandensis *and *B. pseudomallei *infection in *Arabidopsis *plantlets via the roots. The average disease scores were maintained at 1 and increased only slightly at days 6 and 7 and were identical for both *B. thailandensis *and *B. pseudomallei *infection (Fig 5B).

## Discussion

*B. cepacia*, the important opportunistic pathogen often associated with cystic fibrosis and chronic granulomatous disease patients [21], was originally described as a phytopathogen causing soft rot in onions [22]. Subsequently, many strains from various *B. cepacia *complex were shown to be able to cause disease in the alfalfa infection model as well as in the rat agar bead model [23]. In this study, we show that *B. pseudomallei *and *B. thailandensis *are also potential plant pathogens. They are capable of infecting susceptible plants such as tomato.

Plant pathogenic bacteria have been shown to express a large number of T3SS effectors capable of interfering with plant basal defense triggered by bacterial pathogen-associated molecular patterns (PAMPs) as well as Resistance (R) protein-mediated immunity typically characterized by the Hypersensitive Response (HR) [24-26]. The outcome of the interaction with susceptible hosts for these successful pathogens would be disease. We found that the virulence of *B. pseudomallei *in tomato is contributed significantly by T3SS1 and T3SS2, but to a much lesser extent by T3SS3. T3SS1 and T3SS2 are likely non-redundant to each other in causing disease because each mutant demonstrates significant attenuation, possibly because both T3SS1 and T3SS2 are co-ordinately involved in pathogenesis. This is the first time that a role has been defined for T3SS1 and T3SS2 in *B. pseudomallei*, showing that they are functional and not simply vestiges of evolution. The role of T3SS3 could be due to its contribution of a structural component or chaperone to the other two T3SS or an effector which could also interfere with plant cell physiology albeit less efficiently than with mammalian cells. Nevertheless, our study shows the important role played by T3SS in *B. pseudomallei *pathogenesis in tomato plants.

In contrast to tomato, we found that both *B. pseudomallei *and *B. thailandensis *are non-adapted for rice. This is not surprising as *B. pseudomallei *are routinely recovered from rice paddy fields in regions of endemicity such as Thailand and have never been reported to cause any disease in rice plants. It is possible that PAMPs from *B. pseudomallei *and *B. thailandensis *are able to trigger an effective basal defence from rice to halt bacterial colonization, a common means of plant resistance against non-adapted microorganisms [24-26]. Another intriguing possibility is that compounds secreted by rice plants may inhibit the growth of *B. thailandensis *and *B. pseudomallei*. The presence of secondary metabolites induced by *B. pseudomallei *infection in plants with differential susceptibility to disease could reveal novel anti-infective compounds against melioidosis to counter the problem of extensive antibiotic resistance in this bacterium.

Thus, *B. pseudomallei *joins a growing list of human pathogens which have been found to be able to infect plants [27], the first of which to be described was *P. aeruginosa *[28]. The plant host model has been used to perform large scale screening of a library of *P. aeruginosa *mutants to identify novel virulence factors [29] as some virulence factors encoded by genes such as *toxA*, *plcS *and *gacA *were shown to be important for bacterial pathogenesis in both plants and animals [6]. Given the evidence that *B. pseudomallei *T3SS3 may be capable of interacting with both mammalian and plant hosts, and the ability of *B. pseudomallei *to infect tomato, one could develop susceptible plants as alternative host models for large scale screening of *B. pseudomallei *mutants to aid in novel virulence factor discovery, similar to what had been done for *P. aeruginosa*.

Previously, *B. pseudomallei *has been shown to infect *C. elegans *[30] and *Acanthamoeba *species [31] and *C. elegans *could be used as an alternative host model for large scale screening and identification of *B. pseudomallei *virulence factors [30]. Our current finding reveals the additional versatility of *B. pseudomallei *as a pathogen and further research would likely uncover novel bacterial mechanisms capable of interacting with its varied hosts. Much more work is needed to define the susceptibility of various plant species to *B. pseudomallei *to find a suitable plant host for virulence factor discovery. It remains to be seen if *B. pseudomallei *is a natural pathogen for crops such as tomatoes.

## Conclusions

In summary, we identified *B. pseudomallei *as a plant pathogen capable of causing disease in tomato but not rice plants. *B. pseudomallei *T3SS1 and T3SS2 contribute significantly to disease whereas T3SS3 plays a more minor role. Although the significance of *B. pseudomallei *as a natural plant pathogen in the environment is unknown, one could postulate that certain plants may serve as a reservoir for the bacteria. Since *B. pseudomallei *is classified as a bioterrorism agent by the US Centers for Disease Control and Prevention http://www.cdc.gov/od/sap, our findings indicate that it may be necessary to re-evaluate whether *B. pseudomallei *poses threats beyond the animal kingdom and whether plant systems could be used as environmental indicators of the presence of the bacteria either as endemic residents or due to the intentional release by terrorists, a concept that has been previously proposed [27].

## Authors' contributions

YHL participated in the design of the study, carried out most experiments, analyzed and interpreted the data, and performed statistical analysis. YC generated molecular tools and some bacterial mutants. XO performed the TEM. YHG conceived of the study, participated in its design and interpretation of data and wrote the manuscript. All authors read and approved the final manuscript.
